# SaFaRI: sacral nerve stimulation versus the FENIX™ magnetic sphincter augmentation for adult faecal incontinence: a randomised investigation

**DOI:** 10.1007/s00384-015-2492-3

**Published:** 2016-01-12

**Authors:** Annabelle E. Williams, Julie Croft, Vicky Napp, Neil Corrigan, Julia M. Brown, Claire Hulme, Steven R. Brown, Jen Lodge, David Protheroe, David G. Jayne

**Affiliations:** Leeds Institute of Biological and Clinical Sciences, St. James’s University Hospital, Level 7 Clinical Sciences Building, Leeds, LS9 7TF UK; Clinical Trials Research Unit, Leeds Institute of Clinical Trials Research, University of Leeds, Leeds, LS2 9JT UK; Leeds Institute of Health Sciences, University of Leeds, Charles Thackray Building, 101 Clarendon Road,, Leeds, LS2 9LJ UK; Dept. of Colorectal Surgery, University of Sheffield, Northern General Hospital, Sheffield, South Yorkshire S5 7 AU UK; Leeds Primary Healthcare NHS Trust, St Mary’s Hospital, Leeds, LS12 3QE UK; Leeds and York Partnership NHS Foundation Trust, Department of Liaison Psychiatry, Leeds General Infirmary, Great George Street, Leeds, West Yorkshire LS1 3EX UK

**Keywords:** Faecal incontinence, Sacral nerve stimulation, FENIX^TM^, Randomised controlled trial, Surgery

## Abstract

**Purpose:**

Faecal incontinence is a physically, psychologically and socially disabling condition. NICE guidance (2007) recommends surgical intervention, including sacral nerve stimulation (SNS), after failed conservative therapies. The FENIX™ magnetic sphincter augmentation (MSA) device is a novel continence device consisting of a flexible band of interlinked titanium beads with magnetic cores that is placed around the anal canal to augment anal sphincter tone through passive attraction of the beads. Preliminary studies suggest the FENIX™ MSA is safe, but efficacy data is limited. Rigorous evaluation is required prior to widespread adoption.

**Method and design:**

The SaFaRI trial is a National Institute of Health Research (NIHR) Health Technology Assessment (HTA)-funded UK multi-site, parallel group, randomised controlled, unblinded trial that will investigate the use of the FENIX™ MSA, as compared to SNS, for adult faecal incontinence resistant to conservative management. Twenty sites across the UK, experienced in the treatment of faecal incontinence, will recruit 350 patients randomised equally to receive either SNS or FENIX™ MSA. Participants will be followed-up at 2 weeks post-surgery and at 6, 12 and 18months post-randomisation. The primary endpoint is success, as defined by device in use and ≥50 % improvement in the Cleveland Clinic Incontinence Score (CCIS) at 18 months post-randomisation. Secondary endpoints include complications, quality of life and cost effectiveness.

**Discussion:**

SaFaRI will rigorously evaluate a new technology for faecal incontinence, the FENIX™ MSA, allowing its safe and controlled introduction into current clinical practice. These results will inform the future surgical management of adult faecal incontinence.

## Background

Faecal incontinence (FI) is a common and distressing condition with an estimated prevalence of 7.7 % (range 2.0–20.7 %) for the adult population [1]. It is more common in females and with advancing age and is the second most common cause of admission to a nursing home. It impacts on social, physical and mental well-being and is a substantial burden on National Health Service (NHS) resources [2].

Treatment strategies for adult FI are summarised in the National Institute for Health and Care Excellence (NICE) 2007 guidance, which supports the use of sacral nerve stimulation (SNS) for the treatment of adult FI refractory to conservative measures [3]. SNS works by electrical stimulation of the sacral (S2–S4) nerve roots, producing a combination of anal sphincter augmentation and modulation of spinal/supra-spinal pathways. It benefits from a two-stage procedure, which enables the patient to assess acceptability and the clinician to evaluate efficacy prior to permanent implantation. The patient is asked to keep a bowel diary for the 2–3 weeks of temporary stimulation, which allows the clinician to quantify the degree of response. A positive response is defined as a reduction in incontinence episodes or incontinence score of ≥50 % during the stimulation period [4].

SNS has been widely adopted and is currently considered the standard of care for adults with moderate to severe faecal incontinence. Although the short-term efficacy of SNS is good, with 70–80 % of patients experiencing symptom improvement, some 25 % of patients suffer loss of efficacy with time and a further 2–5 % suffer irresolvable complications and undergo explantation [5–7]. From a decision-to-treat perspective, the long-term efficacy is around 50 % [8]. SNS is also very costly. The component costs alone (excluding other direct and indirect medical costs) are £200 for the test stimulation and £9393 for the permanent stimulator. A European trial has calculated the 5-year cumulative costs for SNS at €22,150 per patient, which compared with €33,996 for a colostomy and €3234 for conservative treatment [2]. Despite this, SNS has been shown to be cost-effective. The incremental cost-effectiveness ratio (ICER) for SNS is £25,070 per quality-adjusted life year (QALY) gained, which is within the threshold recommended by NICE as an effective use of NHS resources [9].

Recently, a new device for adult FI has been introduced into clinical practice—the FENIX^TM^ Magnetic Sphincter Augmentation Continence Restoration System (FENIX^TM^ MSA). It consists of a ring of 14 to 20 titanium beads with magnetic cores that are linked together to form an annular structure to be surgically placed around the anal sphincter complex. To defecate, the patient strains in a normal way and the force generated separates the beads to open the anal canal. Continence is restored by means of passive attraction of the beads. The FENIX^TM^ MSA costs £4000. Data on efficacy is limited to a few small single-centre studies [10, 11], a retrospective case-matched comparison to the artificial bowel sphincter where it compared favourably [12], and one small, multicentre, feasibility study [13] that suggests a ≥50 % improvement in continence in 70 % of patients in the short term. However, complications are reported in around 20 % of patients, leading to explantation in around 10 %.

In May 2012, the National Institute for Health Research Horizon Scanning Centre (NIHR HSC) reviewed the evidence on FENIX^TM^ MSA and concluded, “in order to determine its potential place in the pathway of care for FI larger long term studies of the safety, effectiveness and cost-effectiveness of FENIX^TM^ MSA in comparison to existing treatments are needed” [14].

In 2014, the NIHR HTA programme funded the SaFaRI trial—sacral nerve stimulation versus the FENIX™ magnetic sphincter augmentation for adult faecal incontinence: a randomised investigation (Trial Registration: ISRCTN 16077538). The aim of the trial is to undertake a randomised comparison of the FENIX^TM^ MSA as compared to SNS in terms of safety, efficacy, quality of life and cost-effectiveness. This manuscript details the trial design. The results are expected to provide rigorous data on FENIX^TM^ MSA, SNS and a “no treatment” group that fail temporary SNS and are treated by alternative means for the duration of trial recruitment. This will allow healthcare providers to make informed decisions about service provision and facilitate patient choice in the treatment options for FI.

## Methods

### Overall trial aims

The SaFaRI trial will involve a thorough evaluation of the FENIX^TM^ MSA device, as compared to SNS, for the treatment of adult FI. It will evaluate the short-term safety and efficacy of FENIX^TM^ MSA and SNS in adult FI and assess both devices in terms of impact on quality of life and cost-effectiveness.

The primary outcome measure is success, as defined by either FENIX^TM^ MSA or SNS implant in use at 18 months post-randomisation and with a ≥50 % improvement in Cleveland Clinic Incontinence Score (CCIS) [15]. Secondary outcome measures will include the following: length of hospital stay, complications, re-interventions, constipation scores, quality of life and cost-effectiveness.

### Trial sites and participating surgeons

The trial will recruit from at least 20 sites throughout the UK. Participating sites must be an NHS hospital providing specialist treatment for adult FI with experience in the provision of SNS and the facilities to perform endoscopic visualisation of the colorectum, anorectal manometry and endoanal ultrasound. Participating surgeons should be members of The Association of Coloproctology of Great Britain and Ireland (ACPGB&I) and must have experience of a minimum of ten SNS implantations and a minimum of one observed FENIX^TM^ MSA procedure and two FENIX^TM^ MSA procedures under proctorship.

### Trial population

FI is defined as the inability to control the passage of faeces through the anus. For inclusion in the trial, conservative treatments should have been tried and proven ineffective. Both the technology under evaluation (FENIX^TM^ MSA) and the comparator (SNS) will be evaluated on the same patient population. Incontinence may be from any aetiology.

Eligible patients will be aged ≥ 18 years, fit for and willing to undergo either surgical intervention, and able to provide written informed consent. They must have suffered from FI for more than 6 months and experience ≥2 episodes of incontinence per week. They should not have an anal sphincter defect of ≥180° as documented on endoanal ultrasound scan.

Patients will be ineligible for the trial if they have had previous surgical intervention (i.e. failed SNS treatment) for FI, chronic gastrointestinal motility disorders causing diarrhoea, obstructive defaecation symptoms as determined by an obstructed defecation score (OD score) >8, co-existent systemic disease (e.g. scleroderma), active anorectal sepsis, a colorectal cancer diagnosis within 2 years, external rectal prolapse, immunocompromise or known requirement for future magnetic resonance imaging (MRI).

### Trial design

This is a UK multi-site, prospective, parallel group, randomised controlled, unblinded trial to evaluate the safety and efficacy of the FENIX^TM^ MSA for moderate to severe adult FI as compared to SNS. The Clinical Trials Research Unit (CTRU) at the University of Leeds will co-ordinate the trial. The follow-up period finishes 18 months after the last participant is randomised. The University of Leeds is the trial sponsor.

**Fig. 1:**
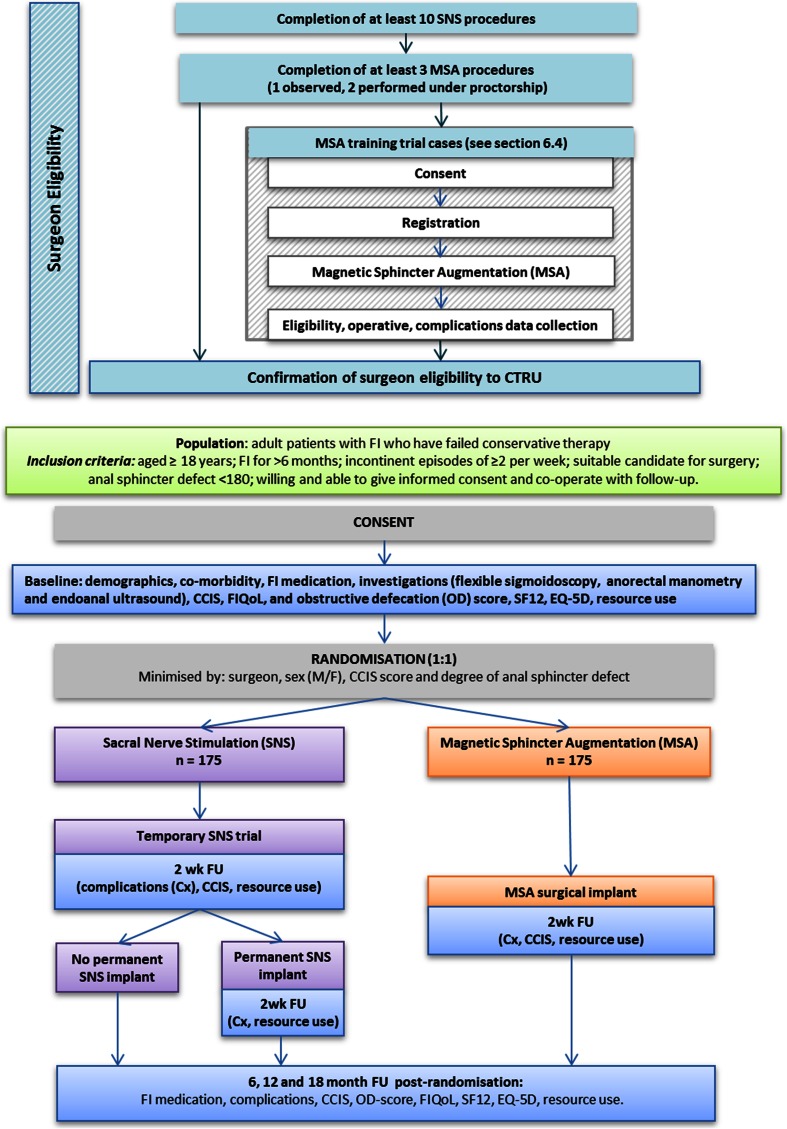
Trial schema

Participants will be randomised on a 1:1 basis to receive either FENIX^TM^ MSA or SNS. A computer-generated minimisation programme that incorporates a random element will be used to ensure treatment groups are well balanced for prognostic factors: treating surgeon, gender, severity of incontinence and degree of anal sphincter defect on endoanal ultrasound. (See Fig. 1: Trial schema).

### Sample size

A total of 350 patients will be recruited with 175 being randomised to each arm.

### Recruitment

Patient recruitment will be over a 30-month period. Baseline investigations will be as per institutional protocol, but must include endoscopic visualisation of the colorectum (flexible sigmoidoscopy as a minimum), anorectal manometry (pudendal nerve testing optional) and endoanal ultrasound. The duration of the trial is expected to be 6 years including setup, recruitment, follow-up and analysis.

### Interventions

The control intervention of SNS implantation will be performed in accordance with each site’s usual practice. SNS implantation may be performed by either implantation of temporary stimulating electrode followed by TINED lead, or straight to TINED lead implant. A period of temporary stimulation is used to assess participant response, which is recorded by means of a 2-week bowel diary. Response is assessed in accordance with each site’s usual practice. The CCIS score will be recorded for trial purposes regardless of how response is assessed locally. If the response is positive (defined as a ≥50 % improvement in incontinence episodes or ≥50 % improvement in CCIS), the participant will proceed to a permanent implant. If the response is negative, the temporary device will be removed and the participant does not receive any further trial intervention but will continue follow-up for the required 18-month follow-up period.

FENIX^TM^ MSA implantation will be standardised in accordance with the manufacturer’s recommendations. Participants will receive a single dose of broad-spectrum intravenous antibiotics at induction of anaesthesia. Implantation of the device will be under fluoroscopic guidance. Participants will be provided with laxatives/stool softeners and analgesics for a period of 7–10 days post-operative.

Should a participant experience failure with either device, which requires explantation, they will not be permitted to undergo implantation of the alternative trial intervention during the 18-month post-randomisation follow-up period. The literature on SNS suggests that around 30 % of patients who undergo a trial of temporary SNS will not have a positive response and will not progress to a permanent implant. Within the trial setting, they will be treated according to current practice, allowing the range of treatments to be captured.

### Follow-up

The planned duration of the trial follow-up is until 18 months after the last participant is randomised. Trial follow-up will include participant review at 2 weeks post-operation and at 6, 12 and 18 months post-randomisation. Participants receiving SNS treatment will be seen at 2 weeks post-temporary implant and if successful 2 weeks post-permanent implant. Any further visits will be according to local clinical practice and will be recorded on the follow-up case report forms (CRFs).

### Data collection and management

Participating sites will record participant data on trial-specific paper CRFs. Clinical data will be collected at baseline, surgery, 2 weeks post-operatively, and at 6, 12 and 18 months post-randomisation. Participant-completed data will be collected at baseline, 2 weeks post-operatively and at 6, 12 and 18 months post-randomisation. .

The CTRU (University of Leeds) will provide overall data and trial management. Received trial data will be monitored for quality and completeness. Missing and discrepant data will be flagged and additional data validations raised as appropriate.

An independent Data Monitoring and Ethics Committee (DMEC) will review the safety and ethics of the trial.

### Outcomes

The primary outcome measure is success, as defined by either FENIX^TM^ MSA or SNS implant in use at 18 months post-randomisation and with a ≥50 % improvement in the CCIS score. Secondary outcome measures will include the following: length of hospital stay, complications, re-interventions, constipation score, quality of life and cost-effectiveness.

### Quality of life

Participants will complete a number of questionnaires designed to capture FI symptoms (CCIS) [15], constipation symptoms (OD score) [16], patient-reported quality of life data (Faecal Incontinence Quality of Life Questionnaire (FIQoL) [17], EQ-5D-5L® [18] and SF-12® [19]) and the costs involved with each treatment, including costs allocated for primary, community and social care services.

Participants will complete all questionnaires at baseline and at 6, 12 and 18 months post-randomisation. In addition, participants will complete the CCIS and the Health and Social Care Resource use Questionnaire 2 weeks post-operatively (only for temporary SNS, and FENIX^TM^ MSA). For the permanent SNS, participants will complete the Health and Social Care Resource use Questionnaire 2 weeks post-operatively.

### Health economic assessment

An economic evaluation will be performed using the perspective of the NHS and social services to aid healthcare providers to make informed decisions about value for money and the future provision of the devices. The objective of the economic evaluation is to identify the within-trial and long-term incremental cost-effectiveness ratios for FENIX^TM^ MSA versus SNS for adult FI. The within-trial economic evaluation will use QALY outcome measures. Quality of life will be measured using the EQ-5D-5L® [20, 21] at baseline and at 6, 12 and 18 months post-randomisation. This will limit the need to interpolate quality of life between observation points and the associated inaccuracy in the estimation of the Health-Related Quality of Life (HRQoL) differences between groups [22]. However, whilst the EQ-5D-5L® is the NICE preferred measure of HRQoL, its sensitivity to detect changes in FI is unproven; we have therefore included the SF-12® as the source of utility data and will undertake a secondary analysis using the SF-12® to derive utility values [23] and present this alongside the EQ-5D-5L® data [24].

NHS resource use associated with each device will be collected either through the CRF (investigations, drugs and referrals for other services), Hospital Episode Statistics (HES) data (inpatient, outpatient and Accident and Emergency) or through a participant questionnaire (contact with primary, community and social care services). Unit costs for health service resources will be obtained from national sources such as the Personal Social Services Research Unit (PSSRU), the British National Formulary (BNF) and NHS Reference cost database. Where national unit costs are not available, the finance departments of NHS Trusts participating in the trial will be asked to provide local cost data. The mean of these costs will be used as the unit cost estimate in the analysis.

The non-parametric bootstrap method will be used to produce a within-trial probabilistic sensitivity analysis of the incremental cost-effectiveness ratio. In addition to presenting the expected incremental cost-effectiveness ratio, we will present the scatterplot on the cost-effectiveness plane, the 95 % cost effectiveness ellipse and the cost-effectiveness acceptability curve [25].

The exact structure and duration of the long-term cost-effectiveness model will be established in discussions with the clinicians on the trial team and after analysis of the complication data observed in the trial. It is likely that the model will be a Markov or semi-Markov state model. As far as possible, the transition rates for the model will be estimated from the clinical trial data. For model parameters for which data cannot be collected within the trial, e.g. long-term outcomes, we will follow the recommended best practice in identifying and synthesising the best available evidence in the literature. The long-term cost-effectiveness modelling will adopt the strategies for addressing issues of perspective and discounting as the within-trial analysis. We will, in addition, undertake an expected value of information analysis.

### Safety evaluation and reporting of adverse events

The term adverse events have been translated into complications for the purpose of safety reporting within the SaFaRI trial. A complication is defined as an untoward medical event in a participant, which has a causal relationship to the trial. The trial includes the surgical intervention directly and any trial-specific interventions. Information on all complications will be collected whether volunteered by the participant, discovered by investigator questioning or detected through physical examination or other investigation.

### Statistical methods

Three hundred and fifty participants will be required to detect at least a 20 % difference in the percentage of successes at 18 months post-randomisation (where success is defined as a device in use and ≥50 % CCIS improvement from baseline) between FENIX^TM^ MSA and SNS at 5 % level of significance, 90 % power, assuming approximately 40 % success on the SNS arm and allowing for 20 % loss to follow-up.

Analyses will be performed on an intention-to-treat (ITT) basis (primary analysis), where participants will be included according to the surgical procedure they were randomised to, and by actual treatment group, where participants will be included according to the surgery actually received (SNS device or FENIX^TM^ MSA device implantation). All hypothesis tests will be two-sided and use a 5 % significance level.

Analyses will exclude training cases, although data collected on training cases will be summarised. Analysis and reporting will be in line with Consolidated Standards of Reporting Trials (CONSORT) guidelines [26]. For the primary analysis, multi-level logistic regression will be used, including adjustment for the factors included in the minimisation algorithm.

Secondary endpoints including SF-12®, EQ-5D-5L®, CCIS and OD-score recorded at baseline and at 6, 12 and 18 months post-randomisation will be analysed using random effects (multi-level) models to account for the hierarchical nature of repeated measures data. The models will include adjustments for minimisation factors, and a categorical covariate will be used to assess the effect of length of time of device in use on these endpoints.

Pattern-mixture multi-level models, which will treat all participant data observed after the removal of their device (explant) as missing data, but also account for the informative nature of the missing data, will be fitted to the secondary endpoints outlined above. Note that this is in contrast to the random effects models outlined above, which incorporate data from participants ‘post-explant’. Therefore, the results yielded by the pattern-mixture multi-level models will act as sensitivity analyses, which can be used to explore the potential issue of disparity in treatment of participants post-explant in each treatment arm.

A subgroup analysis will be performed on participants in the FENIX^TM^ arm in order to explore which potential patients could benefit most from FENIX^TM^. A multi-level logistic regression model will be fitted using the primary endpoint, and the effects of various patient-level covariates (e.g. age, gender, baseline quality of life) on the odds of ‘success’ will be assessed.

Data collected on the safety of FENIX^TM^ MSA and SNS will be analysed using multi-level logistic regression. No formal interim analyses are planned; hence, no statistical testing will take place until final analysis.

### Trial organisation

The SaFaRI trial is funded by the NIHR HTA programme (grant reference 12/35/07). The trial is sponsored by the University of Leeds. Trial supervision will be established according to the principles of Good Clinical Practice (GCP) and in line with the NHS Research Governance Framework (RGF). This will include establishment of a core Project Team, Trial Management Group (TMG), an independent Trial Steering Committee (TSC) and DMEC.

### Ethical considerations

The trial will be conducted in accordance with the principles of GCP in clinical trials, the NHS RGF and through adherence to CTRU SOPs. The trial will operate using the recommendations guiding physicians in biomedical research involving human subjects adopted by the 18th World Medical Assembly, Helsinki, Finland, 1964, amended at the 64th World Medical Association General Assembly, Fortaleza, Brazil, October 2013 [27]. Ethical approval will be sought through NRES. The trial will be submitted to and approved by a REC and the appropriate site-specific assessor for each participating site prior to any recruitment.

## Discussion

New technologies have often been introduced into clinical practice without rigorous evaluation of safety, efficacy and cost-effectiveness. Objective assessment has been overlooked due to the intrinsic appeal of new innovation, the need to be a part of a ‘pioneering group’, or worse, due to the financial incentives from industry. Once introduced, low-grade observational evidence is often used to keep practices going. As a result, it has often been easier to ‘stop them starting’ than to ‘start them stopping’. Ideally, any new technology introduced into clinical practice should be simultaneously evaluated, and in most cases the best way of doing this is by randomised comparison with an already established technique. The SaFaRI trial has been designed to comprehensively achieve a thorough evaluation of the FENIX^TM^ MSA device, as compared to SNS, for the treatment of adult FI, so as to aid healthcare providers to make informed decisions about value for money and future provision of such technology.
